# Exploring dendritic cell based vaccines targeting survivin for the treatment of head and neck cancer patients

**DOI:** 10.1186/1479-5876-11-152

**Published:** 2013-06-20

**Authors:** Annelies W Turksma, Hetty J Bontkes, Janneke J Ruizendaal, Kirsten BJ Scholten, Johanneke Akershoek, Shakila Rampersad, Laura M Moesbergen, Saskia AGM Cillessen, Saskia JAM Santegoets, Tanja D de Gruijl, C René Leemans, Chris JLM Meijer, Erik Hooijberg

**Affiliations:** 1 Department of Pathology, VU University Medical Center-Cancer Center Amsterdam, De Boelelaan 1117, 1081 HV, Amsterdam, The Netherlands; 2 Department of Otolaryngology/Head and Neck Surgery, VU University Medical Center-Cancer Center Amsterdam, De Boelelaan 1117, 1081 HV, Amsterdam, The Netherlands; 3 Department of Medical Oncology, VU University Medical Center-Cancer Center Amsterdam, De Boelelaan 1117, 1081 HV, Amsterdam, The Netherlands; 4 VUMC-CCA, (room 2.26), De Boelelaan 1117, NL-1081 HV, Amsterdam, The Netherlands

**Keywords:** Immunotherapy, DC based vaccines, HNSCC, Survivin, Messenger RNA, Fratricide

## Abstract

**Background:**

New treatment modalities are needed for the treatment of cancers of the head and neck region (HNSCC). Survivin is important for the survival and proliferation of tumor cells and may therefore provide a target for immunotherapy. Here we focused on the *ex vivo* presence and *in vitro* induction of survivin specific T cells.

**Methods:**

Tetramer staining and ELIspot assays were used to document the presence of survivin specific T cells in patient derived material, and to monitor the presence and persistence of survivin specific T cells after repeated *in vitro* stimulation with autologous dendritic cells.

**Results:**

*Ex vivo* analysis showed the presence of survivin-specific T cells in the peripheral blood (by tetramer analysis) and in the draining lymph node (by ELIspot analysis) in a HNSCC and a locally advanced breast cancer patient respectively. However, we were unable to maintain isolated survivin specific T cells for prolonged periods of time. For the *in vitro* generation of survivin specific T cells, monocyte derived DC were electroporated with mRNA encoding full length survivin or a survivin mini-gene together with either IL21 or IL12 mRNA. Western blotting and immunohistochemical staining of dendritic cell cytospin preparations confirmed translation of the full length survivin protein. After repeated stimulation we observed an increase, followed by a decrease, of the number of survivin specific T cells. FACS sorted or limiting dilution cloned survivin specific T cells could not be maintained on feeder mix for prolonged periods of time. Protein expression analysis subsequently showed that activated, but not resting T cells contain survivin protein.

**Conclusions:**

Here we have shown that survivin specific T cells can be detected *ex vivo* in patient derived material. Furthermore, survivin specific T cells can be induced *in vitro* using autologous dendritic cells with enforced expression of survivin and cytokines. However, we were unable to maintain enriched or cloned survivin specific T cells for prolonged periods of time. Endogenous expression of survivin in activated T cells and subsequent fratricide killing might explain our *in vitro* observations. We therefore conclude that survivin, although it is a universal tumor antigen, might not be the ideal target for immunotherapeutic strategies for the treatment of cancer of the head and neck.

## Introduction

Head and neck squamous cell carcinoma (HNSCC) is the sixth most common cancer in the world [1,2]. In the past decades there has been little improvement in the survival rate of HNSCC patients, which is relatively low. Therefore, the development of novel therapies is important. An attractive therapeutic option is immunotherapy, which can be implemented after standard treatment like surgery, radiotherapy or chemotherapy [3-5].

Tumor specific T cells can recognize and kill tumor cells either directly or through the production of cytokines. Dendritic cells loaded with tumor antigens can stimulate T cells leading to T cell activation and expansion. Ideally, tumor associated antigens (TAA) are specific for tumor cells, however, most tumor antigens are also expressed in normal tissues albeit often at lower levels. In a number of cases tumor antigens are derived from proteins that promote transformation and tumor genesis and may even be required to maintain a malignant phenotype [6].

Examples of well-known tumor antigens expressed in HNSCC are p53, Mage-A3, Her2/neu and survivin [7-10]. Survivin is an inhibitor of apoptosis and involved in mitotic spindle formation [11,12]. Survivin is highly expressed in most cancers, but only to a small extent in normal tissue [13]. In normal cells it is expressed in a cell-cycle dependent matter, i.e. only during mitosis, after which it is rapidly degraded [14]. Survivin is a protein that is important for the tumor’s survival and proliferation, and therefore an interesting TAA candidate. Survivin expression in malignant tumors has been associated with poor prognosis [15].

Five splice variants of survivin have been identified: full length or wild type (wt) survivin, survivin 2B, 3B, Dex3 and 2alpha. Survivin wt, 2B and 3B are expressed in the cytoplasm, Dex3 in the nucleus and 2A in the cytoplasm and nucleus. Like survivin wild type, the splice variant Dex3 appears to be anti-apoptotic [16], whereas survivin 2A and 2B may be pro-apoptotic [17-19]. In oropharyngeal tumors and upper urinary tract urothelial carcinoma, nuclear expression of survivin is associated with a poor prognosis [20,21].

In healthy donors survivin specific T cell frequencies in the peripheral blood are often very low, if not undetectable [22]. In cancer patients somewhat higher percentages of survivin specific T cells can be detected in peripheral blood [22]. Survivin specific T cells have been found in several types of tumors, like breast cancer, leukemia, colorectal cancer and melanoma patients [23-28]. According to Weide et al. the absence of survivin specific T cell reactivity in the blood of patients with distant melanoma metastasis is a poor predictor of overall survival, whereas the presence of NY-ESO and Mage-3 specific T cells is a good predictor [29].

In murine models survivin vaccination has been shown to eradicate tumors by inducing survivin specific CD8+ T cells [30-33]. Zeis et al. demonstrated that by using survivin mRNA transfected dendritic cells (DC) to vaccinate mice, long term resistance to lymphoma challenge was obtained. Subsequently they showed that survivin specific T cells were capable of killing tumor cell lines [30], indicating that survivin is immunogenic and could be a useful target for immunotherapeutic strategies.

Moderate results have been reported in phase I/II trials where cancer patients have been vaccinated with synthetic survivin peptides [34-37]. In a phase I trial, myeloma patients received autografts followed by *ex vivo* anti-CD3/anti-CD28 co-stimulated autologous T cells at day 2 after transplantation. Half of the patients additionally received hTERT and survivin peptide immunizations resulting in increased survival compared to the group that did not receive additional vaccination [34]. Unfortunately no distinction was made in the tetramer readout system between survivin and hTERT, therefore the role of survivin in the patients immune response remains unclear. Data obtained from a phase-II peptide vaccination trial in metastatic melanoma showed that survivin-specific T reactivity in about one fifth of the patients correlated with increased overall survival [36]. In a phase I trial the splice variant survivin 2B peptide has been used in HLA-A24 positive patients with oral cancer. Although the response rate was low (one partial responder and nine patients with progressive disease), an increase in survivin specific T cells was observed after vaccination [35].

Here we document the presence of survivin specific T cells in patient derived peripheral blood and lymph nodes and report on our efforts to induce and maintain survivin specific T cells, employing CD8+ T cells and autologous dendritic cells transfected with mRNA encoding survivin in combination with interleukin 12 or interleukin 21.

## Materials and method

### Patient material

Peripheral blood from five HLA-A2 positive HNSCC patients was used to monitor the presence of survivin specific T cells. The HNSCC patients were treated at the VU University Medical Center in Amsterdam, the Netherlands with surgery, chemotherapy, radiotherapy or a combination of these. Blood was drawn at least six weeks after the last treatment via a vena puncture. All patients signed an informed consent form, approved by the Institutional Review Board (METc-VUmc registrationnumber:2009/205). Lymph node derived T cells from a patient suffering from locally advanced breast cancer was used to determine TAA specific T cells by ELIspot. The patient took part in a IRB-approved clinical trial where she received 6 neoadjuvant chemotherapy cycles and signed a written informed consent (METc-VUmc IRB00002991 IORG number 0002436).

### DNA vectors and in vitro transcription of mRNA

Multiple survivin constructs were designed, the survivin inserts were codon modified and generated by Geneart (Regensburg, Germany) and cloned into pGEM4Z vectors (kindly provided by dr. Viggo van Tendeloo, Antwerp, Belgium). The mini-survivin sequence (including the immunodominant HLA-A2 binding T cell epitope) was cloned into pGEM as an EcoRI-NotI fragment, resulting in pGEM-mini-survivin (mini-survivin in short for the mRNA). The resulting open reading frame encoded the following amino-acid-sequence; MQFTE**LTLGEFLKL**DREEEREAEFKSAFTE**LTLGEFLKL**DREEREEERRNKQFTE**LTLGEFLKL**DREEREEERRFNKKQFTE**LTLGEFLKL**DRE*. The Ubi-mini-survivin sequence was cloned into pGEM as an XhoI-NotI fragment, resulting in pGEM-Ubi-mini-survivin (Ubi-mini-survivin in short for the mRNA). The open reading frame encoded the following amino-acid-sequence; MQIFVKTLTGKTITLEVEPSDTIENVKAKIQDKEGIPPDQQRLIFAGKQLEDGRTLSDYNIQKESTLHLVLRLRGVVNSEKFQFTE**LTLGEFLKL**DREEEREAEFKSAFTE**LTLGEFLKL**DREEREEERRNKQFTE**LTLGEFLKL**DREEREEERRFNKKQFTE**LTLGEFLKL**DRE*. The immunodominant HLA-A2 binding T cell epitope survivin(96–104) is indicated in bold in both sequences. The survivin(96–104 epitope is located in the 3rd exon of survivin and is therefore present in wild type survivin and the splice variants 2B and 3B, but absent in survivin splice variants 2alpha and Dex3.

Two full length survivin wild type constructs were used (pGEM-survivin and pGEM-Ubi-survivin along the lines described above. A codon-modified minigene (Ubi-mini-MART for the mRNA) containing four repeats of the altered peptide ligand ELAGIGILTV (was used as described before [38]). *In vitro* transcription was carried out according to manufacturer’s protocol of the T7 mMessage-mMachine kit (Ambion, Huntingdon, Cambridgeshire, UK) to generate m7G(50)pppGcapped IVT mRNA (CAP). mRNA quality was checked by agarose gel electrophoresis. RNA concentration was assayed by spectrophotometrical analysis at OD_260._

### Dendritic cell generation and phenotype analysis

Healthy donor derived peripheral blood mononuclear cells (PBMC) were isolated from HLA-A2 positive buffycoats (Sanquin, Amsterdam, The Netherlands) by density gradient centrifugation using Lymphoprep (Nycomed, Oslo, Norway). Monocytes were isolated by positive selection using a MACS column (MACS; Miltenyi Biotec, Bergisch Gladbach, Germany). PBMCs were labeled with anti-CD14 beads (Miltenyi Biotec) followed by MACS sorting according to the manufacturer’s protocols. Subsequently, the monocytes were cultured with 100 ng/mL recombinant human granulocyte colony stimulating factor (rhGM-CSF; Sagramostim, Berlex) and 10 ng/mL interleukin 4 (IL4; R&D systems) for 6 to 7 days in IMDM medium supplemented with 8% fetal calf serum (FCS; HyClone), 2 mM L-glutamine and antibiotics (100 IE/ml penicillin and 100 μg/mL streptomycin, life technologies). The immature DCs were then matured by culturing them with monocyte conditioned medium (MCM) 25% v/v (generated as previously described [39]) and 50 ng/mL tumor necrosis factor (TNF)α (Miltenyi Biotec). DCs were analyzed phenotypically to determine the maturation status using flow cytometry. The following antibodies were used for phenotypic analysis: IgG1, CD14, CD80, CD83, HLA-DR (BD Biosciences, Heidelberg, Germany), CD1a, CD86, CD40 (Pharmingen, San Diego, CA, USA) labeled with Phycoerythrin (PE). Mean fluorescence index was calculated as follows: MFI-Index = (mean fluorescence intensity marker)/(fluorescence intensity isotype).

### Preparation of dendritic cells for the detection of survivin in western blot and cytospins

Mature DC were electroporated [40] with eGFP, Ubi-survivin or survivin mRNA. Cells were electroporated with 10 μg mRNA in 200 μL electrobuffer (Cell Projects Ltd) in 4 mm cuvets (Cell Projects Ltd) at 300 Volt and 150 μF (Easyject plus, Equibio, Kent, UK). One hour after electroporation cells were washed and plated out in 1*10^6^/mL IMDM medium supplemented with 8% fetal calf serum (FCS; HyClone), 2 mM L-glutamine and antibiotics (100 IE/ml penicillin and 100 μg/mL streptomycin, life technologies).

For Western blot, 4 hours after electroporation with mRNA encoding either eGFP, survivin or Ubi-survivin 1*10^6^ cells were harvested and washed with phosphate-buffered saline (PBS). DC were harvested for cytospin preparations, at time points 2, 4, 6, and 24 hours after electroporation with RNA encoding either eGFP, survivin or Ubi-survivin.

### T cell activation and survivin protein detection

Isolation of resting CD8β positive T cells from HLA-A2 positive, healthy donor PBMC (Sanquin) was performed by positive selection using a MACS column (Miltenyi Biotec). For this purpose, total PBL were incubated with anti-CD8β mAb (clone: 2ST8.5H7, Beckman Coulter, Inc.) and subsequently with microbead-conjugated anti-mouse IgG Abs (Miltenyi Biotec), followed by MACS sorting according to the manufacturer’s protocol. Per donor 6*10^6^ CD8β + T cells were cultured in Yssels medium supplemented with 1% hAB in order to obtain resting T cells. Another aliquot of CD8β + T cells were cultured in the presence of 100 U/mL IL2 and 500 ng/mL PHA in order to obtain activated T cells. After a 48 hour incubation period, the T cells were harvested for FACS and Western blot analysis. T cell activation levels were determined by incubating the T cells with anti-CD25 PE and CD69 FITc (BD Biosciences). Cells were subsequently measured by flow cytometry.

### Western blot analysis

Cell pellets were resuspended in lysis buffer (50 mmol/l Tris/HCl, pH 8.0, 0.5% NP-40, 5 mmol/l EDTA) containing protease inhibitors. Protein levels in the samples were quantified according to manufacturer’s protocol using BCA Protein Assay Kit (Pierce Biotechnology, Rockford, USA). Expression of survivin was detected using a 1:1000 dilution of rabbit-anti-survivin (clone 71G4B7, Cell Signaling Technology, Inc, Boston, USA), followed by incubation with a 1:1000 dilution of polyclonal swine-anti-rabbit immunoglobulin/HRP (DakoCytomation, Glostrup, Denmark). Proteins were visualized with the enhanced chemo-luminescence technique (Amersham Pharmacia Biotech, Piscataway, NJ, USA).

### Cytospins

Cytospin samples of dendritic cells were prepared as followed; per condition 20.000 cells were taken up in 100 uL PBS and spun down for 3 minutes at 600 rpm on a Superfrost plus slide (Thermo Scientific, Braunschweig, Germany). Cytospins were next air dried overnight, fixed in acetone for 10 minutes and stained with anti-survivin(clone 71G4B7, Cell Signalling Technology, Inc.). According to the manufacturer this rabbit mAb was produced by immunizing animals with a synthetic peptide corresponding to residues surrounding cysteine 60 of human survivin and detects endogenous levels of total survivin protein.

### Induction of survivin specific CD8^+^ T cells and detection by tetramer binding

Isolation of resting CD8β positive T cells from healthy donor PBMC (Sanquin) was performed by positive selection using a MACS column (Miltenyi Biotec). For this purpose, total PBL were incubated with anti-CD8β mAb (clone: 2ST8.5H7, Beckman Coulter, Inc., Marseille, France) and subsequently with microbead-conjugated anti-mouse IgG Abs (Miltenyi Biotec), followed by MACS sorting according to the manufacturer’s protocol. Mature DC were electroporated with survivin mRNA in combination with IL12 mRNA or IL21 mRNA as described above. One hour after electroporation DCs were washed and multiple mini-cultures containing 0.5–1 ×10^6^ CD8β T cells, 0.5–1 ×10^5^ mRNA-transfected DCs and 0.25–0.5 ×10^6^ irradiated autologous CD4^+^ T cells were set up in Yssel’s medium supplemented with 1% hAB and 10 U/mL IL7. After 10 days, T cells were analyzed for specificity using PE- and/or APC-labeled HLA-A^*^0201 tetramers (Tm) presenting the survivin 5, 95, 96 or 96 M epitope (survivin 5–14: TLPPAWQPF, survivin 95–104: ELTLGEFLKL, survivin 96–104: LTLGEFLKL, survivin 96 M: L**M**LGEFLKL). We have used in-house prepared tetramers, which at that moment in time could not be tested for specific binding since *bona fide* survivin specific T cell clones were unavailable to us. In a similar case in the past we have validated HPV specific tetramers by using two different fluorescent dyes [41], thus excluding non-specific binding of one of the tetramers. Staining was performed in PBS supplemented with 0.1% BSA and 0.01% Sodium azide for 15 min at 37°C. On day 10 the bulk cultures were re-stimulated with mRNA electroporated DCs and the next day 20 U/mL IL2 (R&D systems, Oxon, UK) was added. Tetramer staining was performed after 7 days and the bulk cultures were re-stimulated weekly as described above.

### ELIspot assay to measure cellular production of IFN- γ

Tumor-draining lymph node material, from patients suffering from locally advanced breast cancer, was used to determine the presence of survivin specific T cells. Antigen-induced interferon gamma (IFN-γ) secretion by survivin induced T cells was measured by an EnzymeLinked Immuno-spot (ELIspot) assay using a modification of a described technique [42]. After wells of nitrocellulose-bottom 96-well plates (MultiScreen-HA; Millipore, Billerica, MA) were coated overnight with 50 μL monoclonal anti-IFN-γ antibody (10 μg/mL; R&D Systems, Minneapolis, MN), the plates were washed, 1% bovine serum albumin was added to each well to block nonspecific binding, and the plates were washed again. The survivin long peptides shown in Additional file 1: Figure S1C were used to load target cells (autologous moDCs). DCs alone or with the survivin long peptides (5 μM) were added in a total volume of 100 μL of complete RPMI medium to the wells and incubated at room temperature for 1 h. The CD4+ or CD8+ T cells were added (1 × 10^4^ cells/well) in 100 μL of complete RPMI medium with added IL2 (20 U/mL) and incubated for 48 h at 37°C with the target cells (1 × 10^3^ cells per well). After the plates were washed, captured IFN-γ was detected by incubation with 50 μL of biotin-conjugated goat anti-human IFN-γ (R&D systems) for 2 h at 37°C in the dark, and the plates were rewashed, incubated for 1 h at 37°C with streptavidin-alkaline phosphatase (Zymed, San Francisco, CA) diluted 1:10.000 in conjugate buffer (1% bovine serum albumin in PBS plus 0.05% Tween 20), washed, and incubated with 50 μL of *FAST* 5-bromo-4-chloro-3-indolylphosphate nitroblue tetrazolium (BCIP/NBT) substrate (Sigma) for 30 min at 37°C in the dark. After drying the plates overnight in the dark, spot-forming cells (SFC) corresponding to individual IFN-γ-secreting cells were counted using an AID ELISPOT reader (Autoimmun Diagnostika GmbH, Strasbourg, Germany). The background number of SFC was determined by incubating the CD8^+^ cells with autologous moDCs alone or autologous moDCs loaded with irrelevant peptide (HPV long peptide), and wells containing CD8^+^ T cells were included as a negative control. Each determination was performed in duplicate, and the results were expressed as SFC/number of plated CD8^+^ T cells.

## Results

### Survivin specific CD8 positive T cells in peripheral blood and lymph nodes from cancer patients

**Figure 1:**
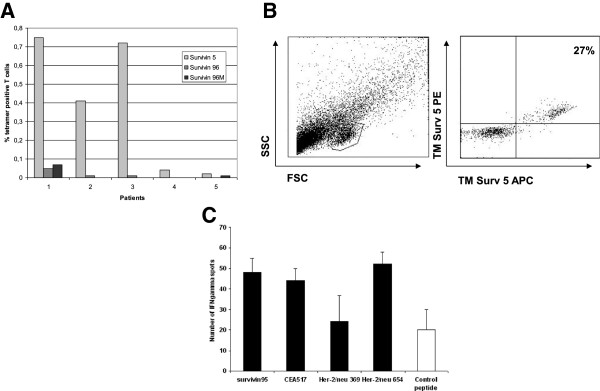
**Spontaneously arising survivin specific T cells in patients. A**) Direct ex vivo tetramer staining of T cells derived from HNSCC patients. The patients (1 thru 5) were all HLA-A2 positive and the CD8 positive T cells were stained with HLA-A2 tetramers containing either the survivin 5 (light grey bars), survivin 96 (dark grey bars) or the modified survivin 96M (black bars) epitope. The percentage tetramer positive T cells in incidated on the Y-axis. **B**) PBMC from HNSCC patients were stained with HLA-A2 tetramers containing the survivin 5 epitope and subsequently sorted by flow cytometry. Cells were plated out at a concentration of 0.2 cells per well and stimulated with feeder mix weekly. The left hand panel shows the forward scatter (X-axis; FSC) and side scatter (Y-axis; SSC) of the cells after four weeks of stimulation with feeder mix. A boxed live gate is indicated. The right hand panel shows tetramer staining of the cells in the live gates. Survivin 5 tetramer labeled with either APC (X-axis) or PE (Y-axis) were used to stain the T cells. The percentage of double tetramer positive cells is shown in the upper right quadrant. **C**) Draining lymph nodes were obtained from a patient with locally advanced breast cancer, who had received neo-adjuvant chemotherapy before surgery. The spontaneously arising T cells were tested in an ELIspot assay against a number of different HLA-A2 binding peptides representing CTL epitopes; Survivin(95–104), CEA(571–579), Her-2/Neu(369–377) and Her-2/Neu(654–662) respectively and HPV16E7-(11–20) peptide as a negative control. Indicated are the number of spots per 100.000 T cells.

We measured the percentage of survivin specific T cells in PBMC derived from the peripheral blood of five patients with head and neck cancer. In three of these patients we found T cells binding HLA-A2 tetramers containing the survivin derived epitope TLPPAWQPFL (Survivin 5–14) (Figure 1A). For one of the patients T cell numbers enabled us to FACS sort the tetramer positive population and clone them by limiting dilution cloning (0.2 cell/well). In 3 out of 20 wells with growing T cells we detected 20-27% survivin specific T cells. A representative example of one of these cultures is given in Figure 1B. However, these three cultures fared poorly (scatter plot shown in Figure 1) and could not be maintained for prolonged periods of time, thus precluding functional analysis, e.g. cytokine production or tumor cell recognition and killing.

In a tumor-draining lymph node obtained from another patient, in this case suffering from locally advanced breast cancer and treated with six cycles of neo-adjuvant chemotherapy, we detected tumor antigen associated specific T cells. The spontaneously arising T cells were tested in an interferon-gamma ELIspot assay against a number of different nine-mer HLA-A2-binding peptides representing tumor associated CTL epitopes; Survivin-95, CEA-571, Her-2/Neu-369 and Her-2/Neu-654. As a negative control we used the virally derived HPV16E7(11–20) peptide. Indicated are the number of spots per 100.000 T cells. The results given in Figure 1C clearly shows survivin specific reactivity amongst reactivity against other TAA.

Having established that spontaneously arising survivin specific T cells can be detected in cancer patients we embarked on *in vitro* induction experiments using healthy donor derived mature DC, electroporated with *in vitro* transcribed mRNA, as stimulator cells and isolated CD8+ T cells as responders, the purpose being to optimize T cell stimulation protocols, employing mature DC, survivin and cytokine encoding mRNA, for *in vivo* vaccination studies in HNSCC cancer patients.

### DNA constructs encoding survivin

**Figure 2:**
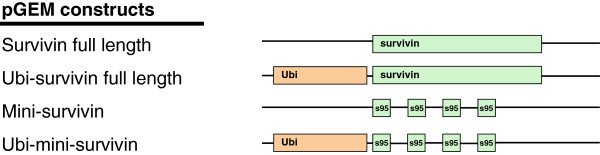
**pGEM constructs containing survivin.** The DNA vector pGEM was used as a template for four different survivin constructs. The green bars indicate the survivin sequence, either full length or the 95–104 sequence. The orange bars indicate part of the ubiquitin sequence.

For the production of *in vitro* transcribed mRNA we designed a number of pGEM constructs encoding either full length survivin or mini-gene constructs containing T cell epitopes derived thereof. A survivin mini-gene construct containing four codon modified repeats of survivin95-107 was prepared as described previously for a different TAA (Mart-1/MelanA) [38]. DC need to process the immunodominant T cell epitope (survivin96-104) from this repeat and present it in the context of HLA-A2. In a number of constructs survivin or the mini-gene repeats were preceded by Ubiquitin sequences to enhance degradation through the proteasome resulting in enhanced MHC class I presentation. An overview of the different constructs, is shown in Figure 2.

### Detection of survivin protein in transfected cells

**Figure 3:**
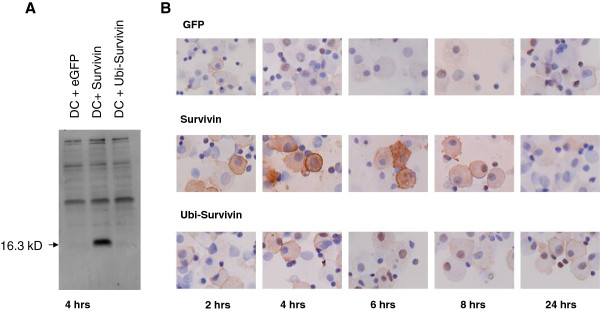
**Survivin expression after electroporation of mRNA in mature dendritic cells. A**) Mature monocyte derived dendritic cells were electroporated with mRNA encoding eGFP, full length survivin or full length Ubi-survivin. Four hours after electroporation the DCs were harvested and lysed. Samples contained equal amounts of cellular protein. The arrow indicates a protein band of 16.3 kD, the predicted molecular weight of full length survivin. **B**) Cytospin preparation were obtained from mature monocyte derived dendritic cells electroporated with mRNA encoding either eGFP (upper panel), full length survivin (middle panel) or full length Ubi-survivin (lower panel). After 2, 4, 6, 8 and 24 hours the electroporated DCs were harvested, spun down on a glass slide and stained with anti-survivin antibodies. Brown staining indicates survivin protein. Nuclei appear in blue.

Next we tested for the presence of survivin protein in transfected cells. Monocyte derived dendritic cells were electroporated with mRNA encoding either GFP as a control, or full length survivin either or not preceded by Ubiquitin sequences. Lysates of cells were prepared four hours after electroporation of mRNA. Western blot analysis showed a clear band for survivin protein, of the expected molecular weight of 16.3 kD, in mature DC electroporated with full length survivin mRNA (Figure 3A, lane 2), but not in control GFP electroporated DC (Figure 3A, lane 1). As expected no specific protein band for survivin was found in the lysate of DC after electroporation with Ubi-survivin mRNA (Figure 3A, lane 3). Staining of high molecular weight protein bands was observed in all three lanes.

Next we determined survivin expression in mature DC by immunohistochemical staining of cytospin preparations (Figure 3B). Cytospins were prepared at 2, 4, 6, 8 and 24 hours after electroporation of mature DC. Low level survivin staining was seen in GFP and in Ubi-survivin electroporated DC at all time points included in the analysis. Staining of survivin protein was most prominent in cytospins taken at 4 h and 6 h after electroporation of mature DC with full length survivin encoding mRNA. Specific staining was reduced to background level in these cells taken at time point 24 h. From these experiments we concluded that the full length survivin sequence, used in two of the DNA constructs shown in Figure 2, is capable of driving the production of survivin protein.

### Induction of antigen specific CD8 positive T cells from healthy donors

Next to the induction of survivin specific T cells we applied our protocols to the induction of Influenza specific (memory) T cells using peptide loaded mature DC, and Mart-1 specific (naive) T cells using mRNA electroporated mature DC [38,41]. CD8+ T cells and monocyte derived mature DC from an HLA-A2 positive healthy donor were used.

**Figure 4:**
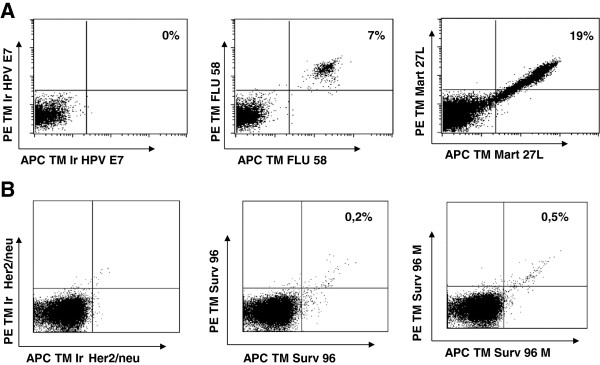
**Tetramer staining after T cell induction. A**) CD8+ T cells from donor 1, were stimulated twice with autologous mature DC loaded with either Influenza peptide or electroporated with mRNA encoding Ubi-Mart4. The T cells were stained with an irrelevant tetramer (HPV E7 11), Influenza 58 and Mart-1 27 L in APC (X-axis) and PE (Y-axis). T cells plotted in the upper right quadrant were considered antigen specific. **B**) CD8+ T cells from donor 2, were stimulated five times with autologous DC electroporated with mRNA encoding full length survivin. The T cells were stained with an irrelevant tetramer (Her2/neu 369), survivin 96 and survivin 96 M in APC (X-axis) and PE (Y-axis). T cells plotted in the upper right quadrant were considered antigen specific.

After *in vitro* stimulation of CD8+ T cells with either peptide loaded (Influenza) or mRNA electroporated (Mart-1) mature DC we tested T cell bulk cultures for the presence of specific T cells employing tetramers. High percentages of specific T cells for Influenza (7% tetramer positive) and Mart-1 (19% tetramer positive) were detected after as little as two *in vitro* stimulations (Figure 4A). In this particular donor the percentage of survivin specific T cells remained below the detection limit. Influenza or Mart-1 specific T cells can be enriched easily by FACS sorting or cloned by limiting dilution cloning. Importantly such T cell clones or cultures can be maintained in culture, using feeder mix stimulations, at near hundred percent purity for prolonged periods of time without loss of specificity or reactivity (data not shown and data published by us previously [38,41,43-45]).

Next we stimulated T cells with mature DC co-electroporated with full length survivin mRNA and IL21 mRNA. After as many as five *in vitro* stimulations we detected low levels of survivin specific T cells staining with tetramers containing either the wild type (0.2% positive for survivin(96–104) or M-modified survivin(96–104) epitope (0.5% positive)). Results are shown in Figure 4B. We noted interferon-γ production as measured in an ELIspot assay upon incubation of T cells with peptide loaded DC for this donor but not for a second donor (see Additional file 1: Figure S1 and Additional file 2: Table S1). After limiting dilution cloning, outgrowth of T cells was found in 69 of 920 wells seeded with survivin tetramer binding T cells obtained after FACS sorting. None of these wells,however, contained viable survivin(96–104) specific T cells.

**Figure 5:**
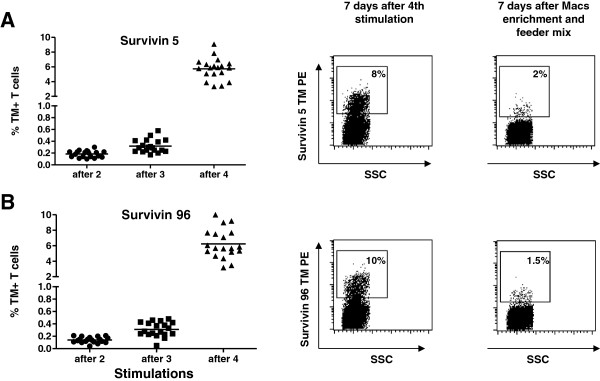
**Detection and MACS enrichment of survivin specific CD8+ T cells.** CD8+ T cells from donor 2, were stimulated three times with autologous DC electroporated with mRNA encoding full length survivin and IL12. The fourth stimulation was performed with mRNA encoding Ubi-survivin and IL12. The T cells were stained with survivin tetramers. Each symbol represents a separate mini-culture. After four stimulations the T cells were MACS sorted en stimulated with feeder-mix. Depicted in the middle panel is the percentage of survivin specific T cells of one representative well after fourth stimulation, just before MACS sorting. In the right hand panel the percentage of MACS sorted survivin specific T cells is depicted after one week on feeder mix. **A**) panels left, middle and right show the data obtained with the survivin 5 epitope. **B**) panels left, middle and right show the data obtained with the survivin 96 epitope.

We next sought to improve on the *in vitro* stimulation of specific T cells by using mRNA encoding Ubi-survivin, mini-survivin of Ubi-mini-survvin in combination with mRNA encoding IL12. Figure 5 shows the results after *in vitro* stimulation of T cells for three times with survivin/IL12 DC, followed by one IVS with Ubi-survivin/IL12 DC. The percentages of tetramer positive T cells are indicated for each well for survivin(5–14) (Figure 5A) and survivin(96–104) (Figure 5B) epitope (range 3-10% positive). Tetramer positive T cells were next MACS-isolated and put in culture on feeder mix. In contrast to our expectations based on prior experience with other T cell specificities, MACS enrichment of survivin specific T cells did not result in a T cell bulk culture with a high percentage of survivin specific T cells (Figure 5A and B right hand panel).

**Figure 6:**
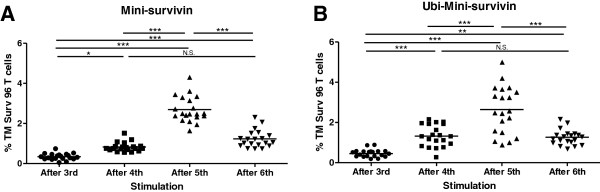
**Induction of survivin specific T cells; appearance and persistence.** CD8+ T cells from an HLA-A2 positive healthy donor (number 6) were primed with autologous mature DC and re-stimulated with JY. Antigen presenting cells were electroporated with mRNA encoding Mini survivin or mini Ubi-survivin. The percentages of survivin 96 tetramer positive T cells found after 3, 4, 5 or 6 rounds of stimulation with APCs electroporated with mRNA encoding either mini-Survivin (panel **A**) or Ubi-mini-survivin (panel **B**) are indicated. Each symbol represents one individual well. **A**) The data points analyzed did not follow a Gaussian distribution and were analysed using a Kruskal-Wallis test with a Dunn’s Multiple Comparison Test. **B**) The data points analyzed followed a Gaussian distribution and were analyzed with a Repeated Measures ANOVA with a Tukey’s Multiple Comparison Test. Statistical analysis was performed and shown as NS: not significant; * = p < 0.05, ** = p < 0.01, *** = p < 0.001.

These results prompted us to perform more T cell inductions and monitor the appearance and persistence of survivin specific T cells more closely. We set up separate mini-cultures of T cells from one donor and stimulated them once with either mini-survivin/IL12 DC or Ubi-mini-survivin/IL12 DC. Subsequent restimulations were performed using B cells (JY), electroporated with mRNA encoding either mini-survivin or Ubi-mini-survivin in combination with mRNA encoding IL12. We set up 20 wells per condition, each indicated with a separate symbol in Figure 6. Prior to *in vitro* stimulation, survivin specific T cells were undetectable using HLA-A2-survivin(96–104) tetramers (data not shown). From the data presented it is clear that the number of tetramer binding T cells went up after the 3rd and 4th *in vitro* stimulation and reached a maximum after the 5^th^ stimulation (range 1-5% positive T cells) in conditions where we used IL12 mRNA in conjunction with either mini-survivin (Figure 6A) or Ubi-mini-survivin (Figure 6B). After the 6th *in vitro* stimulation the numbers went down, never to reach these levels again.

### Survivin protein expression in activated T cells

**Figure 7:**
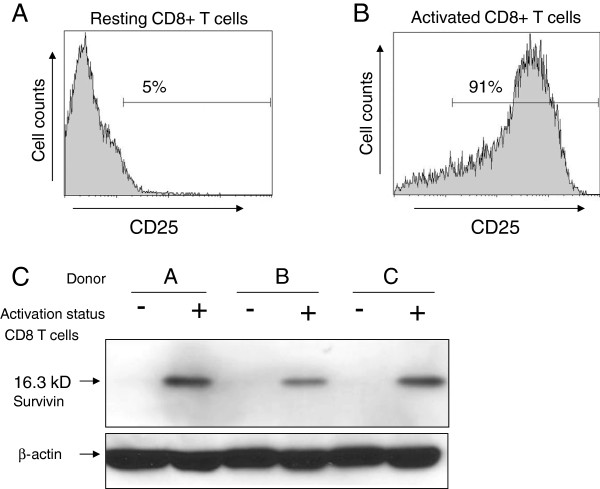
**Protein expression levels of survivin in resting and activated CD8+ T cells.** CD8+ T cells from three healthy donors were isolated and cultured for 48 hours. Half of the T cells per donor were kept in culture medium (resting T cells), the other half were activated with PHA and IL-2 (activated T cells). **A**) Representative FACS histogram of resting T cells stained for the activation marker CD25. About 5% of the plotted cells are CD25 positive. **B**) Representative FACS histogram of activated T cells stained for the activation marker CD25. About 91% of the plotted cells are CD25 positive. **C**) Western blot of resting (−) or activated (+) T cells from three different donors (A, B, C), stained with anti-survivin antibodies. The arrow indicates survivin protein as a band of around 16.3 kD. Household β-actin protein staining was used as a loading control.

Fratricide by survivin specific T cells has been postulated as a possible explanation for the disappearance of survivin expressing T cells in culture. Endogenous expression of survivin by T cells would be a prerequisite for fratricide to take place in the experimental wells. In order to investigate this, we isolated resting and prepared activated T cells from three different healthy donors. Figure 7 shows the expression levels of the T cell activation marker CD25. As expected CD25 expression was low or absent on resting T cells (Figure 7A), whereas approximately 90% of the activated T cells showed high expression levels of CD25 (Figure 7B). Similar data were obtained for another T cell activation marker CD69 (data not shown). Resting and activated T cells were next used to determine endogenous survivin protein expression. From the Western blot data shown in Figure 7C it is clear that activated T cells express appreciable levels of survivin protein, whereas resting T cells do not.

## Discussion

In our efforts to improve treatment of patients with HNSCC we embarked on the induction of T cell responses against widely expressed tumor antigens. One such tumor antigen is survivin which is expressed in almost all tumor types, including HNSCC, and is essential for tumor cell survival. Our laboratories have a substantial track record with respect to the induction, isolation and maintenance of human T cells. In the past we have isolated T cells against a variety of tumor antigens, amongst which Human Papilloma Virus, Mart-1, human telomerase reverse transcriptase (hTERT), ErbB3-binding protein-1 (Ebp1), carcinoembryonic antigen (CEA) and Her-2/neu [41,43-52].

Here we have shown the presence of survivin specific T cells in PBMC of an HNSCC patient by means of tetramer staining (Figure 1A; binding of survivin(5–14): TLPPAWQPF) and in the draining lymph node of a patient with advanced breast cancer by means of ELIspot (Figure 1B; recognition of native survivin95-104: ELTLGEFLKL). It was noted that survivin specific T cells derived from these patients could not be maintained *in vitro* for prolonged periods of times, unlike almost all other T cell clones we obtained in the past. One could argue that such survivin specific T cells may have become senescent due to continuous *in vivo* stimulation by tumor cells present in these patient, resulting in shortening of the telomeric ends of the chromosomes and subsequent clonal exhaustion. Due to low T cell numbers we could not determine the length of the telomeric ends of the chromosomes in these T cells.

This argument of telomere erosion, however, surely does not hold for survivin specific T cells induced from PBMC taken from healthy donor blood (results shown in Figures 4, 5 and 6). Prior to *in vitro* stimulation, these T cells were antigen inexperienced, naive, and therefore will have had long telomeres. The results shown in Figure 6 suggest that survivin specific T cells can only be maintained when the percentage remains below a certain level, which may vary per donor or activation status of the T cells. Another example of this is shown in Figure 5, where we found survivin specific T cells up to 10% of the cells in the life gate of the FACS. However, when these cells were enriched and activated via feeder mix, the percentage of survivin specific T cells dropped dramatically.

A number of research groups have documented the existence of survivin specific T cells, in mice as well as in man. Sorensen et al. published on the generation of an HLA-A2 restricted, survivin(96–104) specific T cell clone [53]. This T cell clone, derived from a breast cancer patient, was able to kill peptide loaded T2-target cells in an HLA-A2 restricted fashion. Moreover the T cell clone was able to recognize and kill a series of HLA-A2 positive tumors cells in classical cytotoxicity assays. In contrast to this, Leisegang et al. reported on the lack of success in generating survivin specific T cell lines or clones employing RNA loaded dendritic cells from HLA-A2 positive donors.

Under normal physiologic conditions survivin is a short lived protein [54]. Endogenous expression of survivin, detected by immunohistochemistry and/or western blot analysis, has been documented in human T cells derived from patients with Multiple Sclerosis or Crohn’s disease [55,56]. Leisegang reported on high level expression of survivin mRNA in activated T cells. Here we have documented the presence of survivin protein in activated CD8+ T cells, and absence thereof in resting T cells (results shown in Figure 7). Since survivin is a protein which is rapidly ubiquitinylated and degraded [54], it would be reasonable to assume that survivin derived protein fragments are also processed and presented in MHC class I, thus making activated T cells an unintended target for survivin specific T cells.

In a commentary, Aqui and Vonderheide pointed out that endogenous expression of potential tumor antigens, like survivin and telomerase, in activated T cells might lead to fratricide killing of T cells [57]. No signs of fratricide however were reported by Sorensen et al., nor did they report on survivin mRNA or protein expression in T cells. Possible explanations could be that their T cell clone carried a mutation in the epitope of survivin(96–104) thereby losing the ability to be presented, or it may have displayed overall malfunctions in HLA-A2 antigen expression, thereby escaping fratricide killing.

Recently, Leisegang et al. showed that *in vitro* cultured, T cell receptor transgenic, survivin(96–104) specific T cells underwent fratricide in an HLA-A2 dependent manner [58]. They used dendritic cells and T cells from HLA-A2 negative donors. DC were electroporated with mRNA encoding full length survivin in combination with HLA-A2 for the induction of HLA-A2 restricted survivin specific T cells. Resulting, HLA-A2 negative, T cells were capable of killing HLA-A2, survivin double positive tumour cells. Introduction of HLA-A2 restricted, survivin specific T cell receptors into polyclonal HLA-A2 positive T cells led to fratricide killing of neighboring T cells [58].

In our experiments we noted that survivin specific T cells could not be maintained in culture for prolonged periods of time. Although we were unable to check for fratricide mediated killing of neighboring T cells, due to a lack of sufficient T cell numbers, our findings can very well be explained by the observations made by Leisegang et al. for the immunodominant T cell epitope survivin(96–104). Although speculative, we documented lack of T cell outgrowth for the survivin(5–14) epitope (TLPPAWQPF) shown in Figure 1 as well.

Fratricide amongst survivin specific CD8+ T cells has now been documented [58]. It remains unclear whether fratricide will also occur in CD4+ T cells on the basis of survivin epitopes presented in MHC-class II [59,60]. A number of different splice variants have been described for survivin [16-19]. Whether all of these are expressed in activated T cells remains to be elucidated. One could envision that T cell reactivity directed against survivin splice variants, expressed in tumor cells but not in T cells, may still be useful in cancer immunotherapy approaches [61,62]. This notion may be exemplified by the HLA-A24 restricted survivin 2B epitope 80–88 generated by Takahasi and colleagues [63]. The isolation of HNSCC tumor infiltrating T cells and the identification of epitopes being recognized may offer new therapeutic targets. Alternatively, clinical use of *ex vivo* expanded tumor infiltrating T cells with a range of (unknown) specificities may also provide a viable treatment option for HNSCC patients [64]. Survivin as an immunotherapeutic target may still be useful in DC based active immunotherapy, since *in vivo* the percentages of survivin specific T cells will most likely remain below the ‘fratricide threshold’. However for adoptive transfer purposes the use of survivin specific T cells is self-destructive and therefore not feasible.

## Competing interests

The authors declare that they have no competing interests.

## Authors’ contributions

AWT performed experiments, analyzed the data and co-wrote the manuscript; HJB co-wrote the project underlying this manuscript, interpreted the data and co-wrote the manuscript; JJR, KBJS, JA, SR, LMM, SAGMC and SJAMS performed experiments and analyzed the data; SJAMS and TDdG co-wrote the manuscript and provided materials and intellectual input; CRL and CJLM co-wrote the project underlying this manuscript and co-wrote the manuscript; EH wrote the project underlying this manuscript, analyzed and interpreted the data, supervised the project and execution of the experiments, wrote the manuscript and the rebuttal to reviewers comments. All authors read and approved the final manuscript.

## Supplementary Material

Additional file 1: Figure S1 **A-B)** T cells stimulated with survivin/IL21-DC from two donors were tested in an IFN-y ELIspot assay. Autologous mature DC loaded with survivin overlapping long-peptides were used as antigen presenting cells. The long peptides were pooled in four different mixes; Mix A, B, C and D (details in Figure S1C). The number of spots against the mixes A-D and of the irrelevant HPV long peptide are shown on the Y-axis. On the X-axis the number of spots per well are indicated. **A)** The number of INF-y spots for donor 2. **B)** The number of INF-y spots for donor 3. **C)** The aminoacid sequence of full-length wild-type survivin is depicted. The long-peptides grouped into mix A,B,C and D used in the ELIspot assay are depicted underneath. Click here for file

Additional file 2: Table S1 Overview of survivin specific T cell inductions per donor. Shown are the mRNA constructs, the number of CD8+ T cells, the antigen presenting cell (for initial priming and subsequent restimulation), and the readout systems (tetramer and/or ELIspot) used. Click here for file
