# Supplementary material for: Exploring dendritic cell based vaccines targeting survivin for the treatment of head and neck cancer patients
Source: J Transl Med. 2013 Jun 20;11:152. doi: 10.1186/1479-5876-11-152 (PMC3695847; doi:10.1186/1479-5876-11-152)
Supplement: Additional file 1: Figure S1 — A-B) T cells stimulated with survivin/IL21-DC from two donors were tested in an IFN-y ELIspot assay. Autologous mature DC loaded with survivin overlapping long-peptides were used as antigen presenting cells. The long peptides were pooled in four different mixes; Mix A, B, C and D (details in Figure S1C). The number of spots against the mixes A-D and of the irrelevant HPV long peptide are shown on the Y-axis. On the X-axis the number of spots per well are indicated. A) The number of INF-y spots for donor 2. B) The number of INF-y spots for donor 3. C) The aminoacid sequence of full-length wild-type survivin is depicted. The long-peptides grouped into mix A,B,C and D used in the ELIspot assay are depicted underneath. [file 1479-5876-11-152-S1.pptx]

## Slide 1
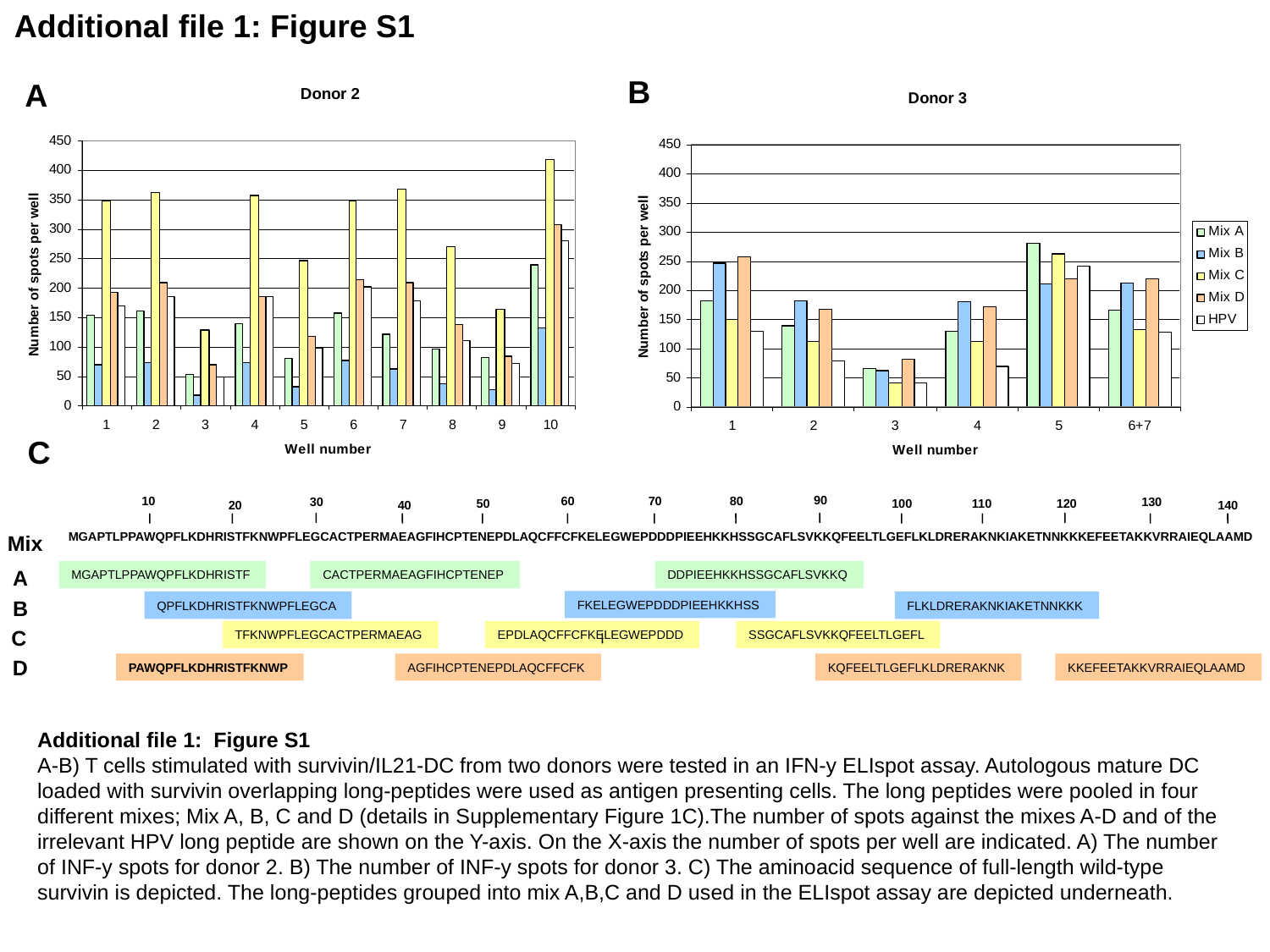

Additional file 1: Figure S1
B
A
C
90
10
60
70
80
30
130
50
120
100
110
20
40
140
MGAPTLPPAWQPFLKDHRISTFKNWPFLEGCACTPERMAEAGFIHCPTENEPDLAQCFFCFKELEGWEPDDDPIEEHKKHSSGCAFLSVKKQFEELTLGEFLKLDRERAKNKIAKETNNKKKEFEETAKKVRRAIEQLAAMD
Mix
A
MGAPTLPPAWQPFLKDHRISTF
CACTPERMAEAGFIHCPTENEP
DDPIEEHKKHSSGCAFLSVKKQ
B
FKELEGWEPDDDPIEEHKKHSS
QPFLKDHRISTFKNWPFLEGCA
FLKLDRERAKNKIAKETNNKKK
C
TFKNWPFLEGCACTPERMAEAG
EPDLAQCFFCFKELEGWEPDDD
SSGCAFLSVKKQFEELTLGEFL
D
PAWQPFLKDHRISTFKNWP
AGFIHCPTENEPDLAQCFFCFK
KQFEELTLGEFLKLDRERAKNK
KKEFEETAKKVRRAIEQLAAMD
Additional file 1: Figure S1
A-B) T cells stimulated with survivin/IL21-DC from two donors were tested in an IFN-y ELIspot assay. Autologous mature DC loaded with survivin overlapping long-peptides were used as antigen presenting cells. The long peptides were pooled in four different mixes; Mix A, B, C and D (details in Supplementary Figure 1C).The number of spots against the mixes A-D and of the irrelevant HPV long peptide are shown on the Y-axis. On the X-axis the number of spots per well are indicated. A) The number of INF-y spots for donor 2. B) The number of INF-y spots for donor 3. C) The aminoacid sequence of full-length wild-type survivin is depicted. The long-peptides grouped into mix A,B,C and D used in the ELIspot assay are depicted underneath.
