# Supplementary material for: Exploring dendritic cell based vaccines targeting survivin for the treatment of head and neck cancer patients
Source: J Transl Med. 2013 Jun 20;11:152. doi: 10.1186/1479-5876-11-152 (PMC3695847; doi:10.1186/1479-5876-11-152)
Supplement: Additional file 2: Table S1 — Overview of survivin specific T cell inductions per donor. Shown are the mRNA constructs, the number of CD8+ T cells, the antigen presenting cell (for initial priming and subsequent restimulation), and the readout systems (tetramer and/or ELIspot) used. [file 1479-5876-11-152-S2.pptx]

## Slide 1
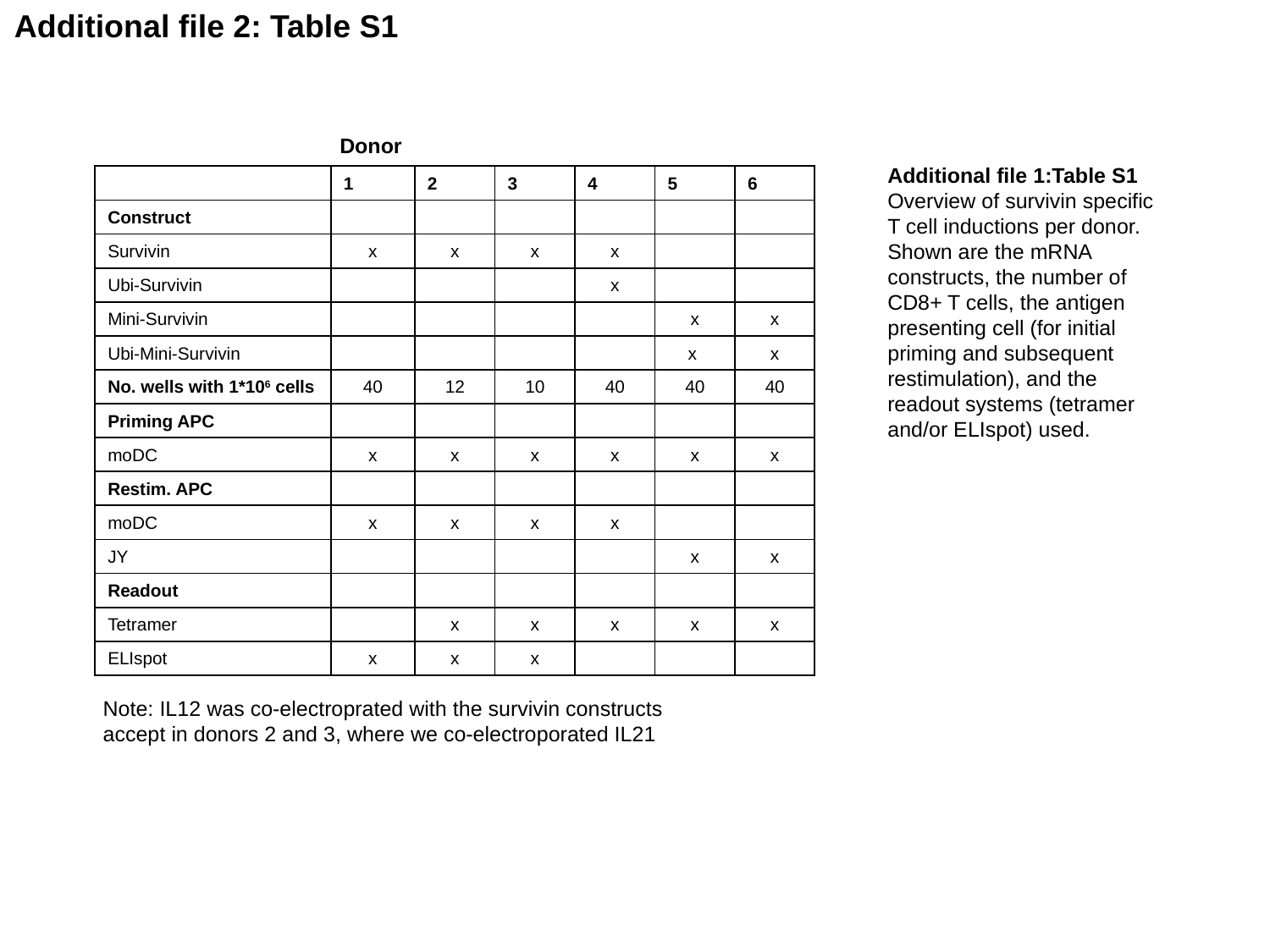

Additional file 2: Table S1
Donor
Additional file 1:Table S1
Overview of survivin specific T cell inductions per donor. Shown are the mRNA constructs, the number of CD8+ T cells, the antigen presenting cell (for initial priming and subsequent restimulation), and the readout systems (tetramer and/or ELIspot) used.
| | 1 | 2 | 3 | 4 | 5 | 6 |
| --- | --- | --- | --- | --- | --- | --- |
| Construct | | | | | | |
| Survivin | x | x | x | x | | |
| Ubi-Survivin | | | | x | | |
| Mini-Survivin | | | | | x | x |
| Ubi-Mini-Survivin | | | | | x | x |
| No. wells with 1\*106 cells | 40 | 12 | 10 | 40 | 40 | 40 |
| Priming APC | | | | | | |
| moDC | x | x | x | x | x | x |
| Restim. APC | | | | | | |
| moDC | x | x | x | x | | |
| JY | | | | | x | x |
| Readout | | | | | | |
| Tetramer | | x | x | x | x | x |
| ELIspot | x | x | x | | | |
Note: IL12 was co-electroprated with the survivin constructs
accept in donors 2 and 3, where we co-electroporated IL21
